# A Retrospective Study on the Etiological Factors of Orofacial Pain in a Malaysian Sample

**DOI:** 10.1055/s-0041-1735443

**Published:** 2021-11-09

**Authors:** Nazih Shaban Mustafa, Muhannad Ali Kashmoola, Basma Ezzat Mustafa Al-Ahmad, Mardhiah Abidah Binti Hazman Fansuri, Nur Hazwani Mohamad Jurimi, Sayfaldeen Kashmoola

**Affiliations:** 1 Department of Oral Maxillofacial Surgery and Oral Diagnosis, Faculty of Dentistry, International Islamic University Malaysia, Kuantan, Pahang, Malaysia; 2 Department of Fundamental Dental and Medical Sciences, Kulliyyah of Dentistry, International Islamic University Malaysia, Kuantan, Pahang, Malaysia; 3 Oral Health Division, Ministry of Health, Kuala Lipis, Pahang, Malaysia; 4 Oral Health Division, Ministry of Health, Kadut, Sabah, Malaysia; 5 Department of Prosthodontics, Faculty of Dentistry, Lincoln University College, Petaling Jaya, Selangor, Malaysia

**Keywords:** etiological factors, orofacial pain, Malaysian

## Abstract

**Objective** This study aimed to examine the etiological factors of orofacial pain for patients attending dental clinic at Faculty of Dentistry, International Islamic University Malaysia (IIUM).

**Materials and Methods** This retrospective study examined the data of 248 patients who have attended dental clinic at Faculty of Dentistry IIUM and suffering from different types of orofacial pain. The data were collected from January 2010 to November 2018. The etiologies of pain were classified according to International Classification of Orofacial Pain, 1st edition (2020).

**Statistical Analysis** The association of age and gender with orofacial pain was evaluated by using the Chi-square test, and the significance level was set to 0.05.

**Results** Collected data showed that orofacial pain has different etiologies among the patients attending the dental clinic at Faculty of Dentistry IIUM. Moreover, a statistically significant relation was observed between orofacial pain toward gender and different age group.

**Conclusion** The findings proposed that the orofacial pain has a variety of etiological factors with the highest percentage of orofacial pain attributed to disorders of dentoalveolar and anatomically related structures among patients attending dental clinic at Faculty of Dentistry IIUM.

## Introduction

Pain is a crucial protective mechanism of a person's body. It is defined as unpleasant sensory and emotional experiences associated with the actual or possible tissue damage. Therefore, pain gives impact to someone's decision for seeking a treatment. 1

Orofacial pain is a common complaint among patients attending dental clinic. It has been linked to pain that originates from oral structures, which come together with facial pain and comprises of region below the orbitomeatal line and above the neck and anterior to the ears. Nevertheless, radiating pain from one area to another might rise as craniofacial region has high density of anatomical structures. Hence, a comprehensive history and investigation of patient need to be executed by the dentists or physicians to obtain relevant and accurate etiological factors of that pain is essential to provide proper management. This shows that orofacial pain is not straightforward to be diagnosed since it is very broad. 2

Etiology of pain can be due to various reasons such as an early warning physiological protective system (nociceptive pain). Besides, pain also occurs after unavoidable tissue damage which assists healing by activation of the immune system (inflammatory pain). In other cases, abnormal functioning of the nervous system can also cause pain. Regarding this matter, pain can be due to disease state of the nervous system that can occur after nervous system damage (neuropathic pain) or dysfunction of the nervous system without any damage of inflammation (dysfunctional pain). 3

The objective of this study is to recognize the etiology of pain for patients attending IIUM dental clinic and its association with gender and age, on top of that to highlight types of pain in Malaysian sample which will be as a reference and base line for future studies.

## Materials and Methods

Prior to commencement of this study, ethical approval was obtained from Research Ethics Committee, International Islamic University of Malaysia (IREC, ID no.: IREC 2018–047).

- In this retrospective study, a total number of 248 clinical records for patients attended the IIUM Dental clinic for the period of eight years from January 2010 to November 2018 were selected. Inclusion criteria are as follows:
- Patients attending IIUM dental clinics with pain as chief complaint
- Patients aged 17 years old and above

Patient's gender, age, and diagnosis were recorded in a special sheet. Data were assessed and classified according to International Classification of Orofacial Pain, 1st edition (ICOP) 2020. 4

Statistical analysis was performed by using SPSS for Windows, version 23 (IBM Corp. Released 2015. IBM SPSS Statistics for Windows, Version 23.0. Armonk, New York, United States: IBM Corp.). The significance level was set to 0.05. The descriptive statistics, including numbers, percentages (for categorical variables), means, and standard deviations (for numerical data), were used to examine the study sample. Chi-square test of independence was used to evaluate the association of age and gender with orofacial pain.

## Results

### Patients

Among all 248 recorded clinical cases with the complaint of orofacial pain, 223 (89.91%) reported as orofacial pain attributed to disorders of dentoalveolar and anatomically related structures, 23 (9.27%) with temporomandibular joint disorders (TMD), and two (0.8%) idiopathic orofacial pain.

### Gender and Age

A total of 111 (44.76%) males and 137 (55.24%) females were included in this study. There is no statistically significant difference between females and males in relation to orofacial pain ( *p* = 0.327).

**Fig. 1:**
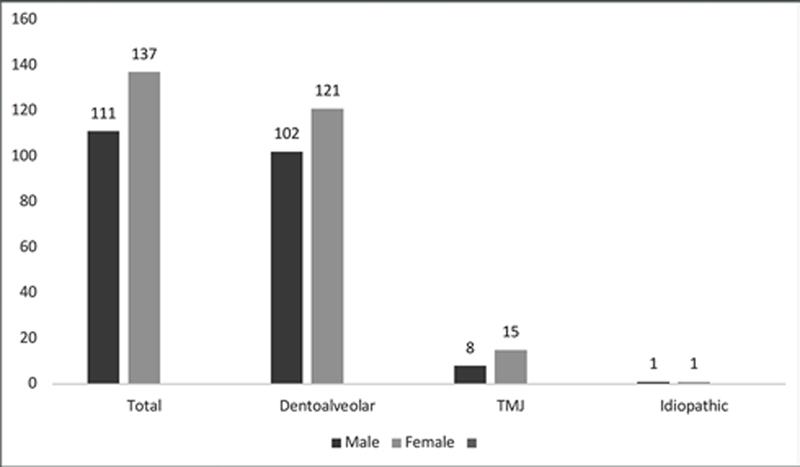
Distribution of orofacial pain ( *n* ) according to gender.

Fig. 1 shows the gender in relation to orofacial pain which are classified into:

- Orofacial pain attributed to disorders of dentoalveolar and anatomically related structures
- Temporomandibular joint disorders
- Idiopathic orofacial pain

**Fig. 2:**
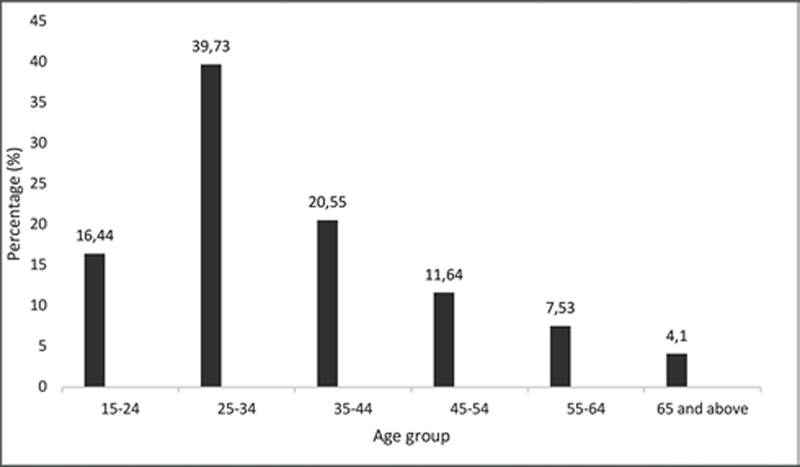
Distribution of orofacial pain (%) according to age groups.

Patients' age ranged from 17 to 84 years old, with the mean age 33.50 ± 13.21 years. Fig. 2 shows age groups distribution according to the orofacial pain. Statistically, orofacial pain dependent toward different age groups ( *p* = 0.001).

### Diagnosis

Tables 1 and 2 comprised of three major different types of orofacial pain in relation to age and gender, respectively. The tables show the details of 223 recorded orofacial pain attributed to disorders of dentoalveolar and anatomically related structures such as dental abscess, pulpitis, pericoronitis, dry socket, tooth sensitivity ulcer, and cheilitis. Meanwhile TMD presented with 23, while idiopathic orofacial pain presented as two burning mouth syndrome (BMS).

**Table 1:** Distribution of etiologies of orofacial pain on different age groups

Type of pain	Age groups (y)						Total
	>25	25–34	35–44	45–54	55–64	64<	
Orofacial pain attributed to disorders of dentoalveolar and anatomically related structures	41	78	31	20	11	6	223
Dental abscess	3	13	4	4	2	1	27
Irreversible pulpitis	9	20	11	4	2	0	46
Reversible pulpitis	11	24	9	6	6	4	60
Tooth sensitivity	1	1	6	3	1	1	13
Pericoronitis	17	20	0	3	0	0	40
Dry socket	0	0	1	0	0	0	1
Ulcer	20	9	1	1	3	1	35
Cheilitis	1	0	0	0	0	0	1
Temporomandibular joint disorder	10	6	1	3	2	1	23
Idiopathic orofacial pain/Burning mouth syndrome	0	1	1	0	0	0	2
Total							248

**Table 2:** Distribution of etiologies of orofacial pain among gender of patients

Type of pain	Gender		*n*
	Male	Female	
Orofacial pain attributed to disorders of dentoalveolar and anatomically related structures	102	121	223
Dental abscess	12	15	27
Irreversible pulpitis	22	24	46
Reversible pulpitis	30	30	60
Tooth sensitivity	8	5	13
Pericoronitis	14	26	40
Dry socket	1	0	1
Ulcer (Aphthous)	8	4	12
Cheilitis	0	1	1
Temporomandibular joint disorders TMD	8	15	23
Idiopathic orofacial pain/burning mouth syndrome	1	1	2

Abbreviation: TMD, temporomandibular joint disorder.

## Discussion

Disorders of dentoalveolar and anatomically related structures presented with localized, acute, and sharp pain which comes in short duration, as an example of reversible and irreversible pulpitis. Such pain forces a lot of patients to come and see the dentist immediately to relieve the pain. This interpretation explains the results of this study, which showed that pulpal inflammation was the highest etiologic factor of patients attending IIUM dental clinic. The results are in accordance with the study done by Renton et al. 5 The second highest number of the patients with orofacial pain attributed to disorders of dentoalveolar structures was pericoronitis, which is in accordance with the study conducted by Bamidele 6 and Yilmaz et al. 7 In this study, dental abscess shows high number among the etiological factor of orofacial pain that arises from the tooth, which is similar to Boeira et al 8 and Robertson et al. 9 In contrary to the present study, Siddiqui et al reported that tooth sensitivity is found to have high numbers of patients with dentoalveolar origin of orofacial pain. 10 Among the other causes of pain, oral ulceration showed the highest etiological factors in the present study, which is consistent with research conducted by Ahmed and Uddin, 11 who demonstrated that ulcers occupy the most common oral mucosal lesions out of the worldwide population. 11

Pain arises from TMD origin may present as acute or chronic pain like in regularly presented with dull pain. Because of the nature of the pain, it makes the number of patients who seek for a treatment to decrease. 12 Prolonged mouth opening is often related to the most frequent form of acute onset of pain in TMD, which can happen after dental treatment or trauma and many other causes. Otherwise, TMD that come with chronic onset of pain often associated with myalgia of masticatory muscles secondary to clenching habits in such patient with nocturnal bruxism. 12 This variation of onset of pain caused the increases number of people who seek treatment, and this has been shown in the present study where TMD was the second most common etiology of orofacial pain.

Diagnosis and treatment of chronic orofacial pain are one of the most challenging procedures in dentistry. 13 This difficulty arises from the complexity in identification of etiological factors due to ambiguous presentation of signs and symptoms. Moreover, the financial burden and time consumed for diagnosis of pain and reviews of patient, thus making this process frustrating for both patient and dentist. 14 As the perception of pain is subjective and interpreted differently, patients with orofacial pain may come with various complaints. Until recently, no certain classification of orofacial pain that can be followed. Most common classes that have been used are topographical (odontogenic vs. nonodontogenic) and/or chronological (chronic vs. acute). 15 In year 2020, International Headache Society released the first ICOP. 4 In this study, ICOP was used for the classification of pain.

The findings of this study propound that the orofacial pain has variety of common etiologies among patients attending dental clinic at Faculty of Dentistry IIUM. They are often presented with signs and symptoms that mimic one another, which may confuse the clinician to differentiate and reach definite diagnosis, to give the proper treatment plan. Due to the resembling signs and symptoms of orofacial pain with different contributing disorders, the dentist needs to follow the rule of “common things are common.” Therefore, disorders of dentoalveolar and other supported structures (reversible pulpitis, irreversible pulpitis, pulpal infection, pulpal necrosis, periapical or periodontal abscess, cracked tooth syndromes, pericoronitis, alveolar osteitis, and dental trauma) should always be considered first prior to consideration of other etiological factors or disorders. Thus, thorough clinical examination of the dentoalveolar complex should be performed. 5 12 16 17 18

Orofacial pain appeared to be one of the main complaints of patients attending dental clinic which is in accord with the studies done by Zakrzewska, 12 Horst et al, 19 and Rezazadeh et al. 20 The results of this study also showed that there were different etiologies of orofacial pain. Most of the patients came with orofacial pain attributed to disorders of dentoalveolar and anatomically related structures compared with other types of pain origin.

The effect of gender and age on the frequency of orofacial pain among patients attending dental clinic Faculty of Dentistry of IIUM was explored in this study. In terms of numbers and percentages, females constituted a slightly higher proportion compared with males. Additionally, higher number of female patients had dental abscess, pulpitis, pericoronitis, TMD, and ulcer. Those findings are in contrast to study done by Smiljic et al 21 and Aggarwal et al. 22 However, the present study showed that there was statistically no significant difference between genders in relation to orofacial pain, which is in line with Rikmasari et al. 23 In addition, the number of females was more than males in pericoronitis which is similar to the studies performed by Yamalik and Bozkaya 24 and LeResche. 25

There was a statistically significant association between 25 and 34 years' age group and orofacial pain, which is in agreement with Pau et al 26 and Kurchid et al. 27 Pain originated from dentoalveolar and supporting structures contributed to the highest numbers of patients with age group of 25 to 34 years old compared with other types of orofacial pain. Among this type of orofacial pain, pulpitis and pericoronitis are the commonest form of dental condition in this age group that cause patients to seek for a treatment. This is consistent with the studies performed by Kakoei et al 28 and Singh et al. 29 Moreover, this finding is in line with study was performed by Al-Naaimi et al. 30

Various types of chronic orofacial pain of unknown origin exist. The new ICOP is the first comprehensive classification that uniquely deals with orofacial pain. The ICOP is a hierarchical classification modeled on the International Classification of Headache Disorders and covers pain in dentoalveolar and anatomically related tissues, muscle pain, temporomandibular joint pain, neuropathic pain affecting cranial nerves, pain resembling primary headaches, and idiopathic pain in the orofacial region. 31

The pathologies associated with these conditions, which include BMS and persistent idiopathic facial pain, remain unclear. Recently, it was hypothesized that these conditions may represent neuropathic pain. Although they may be caused by peripheral sensitization, central sensitization, or impaired upper central nervous control function. In this study, there were only two cases one female and one male with history of BMS. It is challenging to achieve the diagnosis and management for such condition.

## Conclusion

This study concluded that patients attending dental clinic Faculty of Dentistry IIUM have different etiological factors of orofacial pain, with the highest percentage of orofacial pain attributed to disorders of dentoalveolar and anatomically related structures. The findings of this study can help in recognizing the etiology of pain for patients and hence provide a better treatment plan and management in the future.
